# Dysregulation of the Nitric Oxide/Dimethylarginine Pathway in Hypoxic Pulmonary Vasoconstriction—Molecular Mechanisms and Clinical Significance

**DOI:** 10.3389/fmed.2022.835481

**Published:** 2022-02-17

**Authors:** Juliane Hannemann, Rainer Böger

**Affiliations:** ^1^Institute of Clinical Pharmacology and Toxicology, University Medical Center Hamburg-Eppendorf, Hamburg, Germany; ^2^Institute DECIPHER, German-Chilean Institute for Research on Pulmonary Hypoxia and its Health Sequelae, Hamburg, Germany

**Keywords:** high altitude, endothelium/physiopathology, asymmetric dimethylarginine (ADMA), hypoxaemia, chronic obstructive lung disease (COPD), obstructive sleep apnea syndrome (OSAS)

## Abstract

The pulmonary circulation responds to hypoxia with vasoconstriction, a mechanism that helps to adapt to short-lived hypoxic episodes. When sustained, hypoxic pulmonary vasoconstriction (HPV) may become deleterious, causing right ventricular hypertrophy and failure, and contributing to morbidity and mortality in the late stages of several chronic pulmonary diseases. Nitric oxide (NO) is an important endothelial vasodilator. Its release is regulated, amongst other mechanisms, by the presence of endogenous inhibitors like asymmetric dimethylarginine (ADMA). Evidence has accumulated in recent years that elevated ADMA may be implicated in the pathogenesis of HPV and in its clinical sequelae, like pulmonary arterial hypertension (PAH). PAH is one phenotypic trait in experimental models with disrupted ADMA metabolism. In high altitude, elevation of ADMA occurs during long-term exposure to chronic or chronic intermittent hypobaric hypoxia; ADMA is significantly associated with high altitude pulmonary hypertension. High ADMA concentration was also reported in patients with chronic obstructive lung disease, obstructive sleep apnoea syndrome, and overlap syndrome, suggesting a pathophysiological role for ADMA-mediated impairment of endothelium-dependent, NO-mediated pulmonary vasodilation in these clinically relevant conditions. Improved understanding of the molecular (dys-)regulation of pathways controlling ADMA concentration may help to dissect the pathophysiology and find novel therapeutic options for these diseases.

## Introduction

Hypoxia is a deadly threat to every cell and to the organism as a whole. It is therefore not surprising that complex molecular mechanisms have evolved that help the cell to maintain its integrity during short-lived periods of hypoxia, as well as physiological mechanisms that help the organism to adapt to conditions of low oxygen supply.

In most organs, the response to a mismatch between oxygen demand and supply is an increase in blood flow. This has been demonstrated for the coronary, cerebral, renal, and other vascular beds (1–3). Hypoxia in the systemic circulation may result from local vascular occlusion (either by vasospasm or thromboembolism), low oxygen delivery with the blood stream (either because of anemia or reduced arterial hemoglobin oxygen content), or reduced perfusion volume (e.g., in chronic heart failure). In each case, compensatory mechanisms aiming at increasing local blood flow are activated to minimize ischemic tissue damage. Recurrent brief periods of ischemia in the systemic circulation activate mechanisms leading to improved protection of tissues from ischemic cell death. This interesting phenomenon called ischemic pre-conditioning has been extensively investigated and reviewed (4–6); further detailed description is beyond the scope of this review.

By contrast, the vast majority of tissue oxygen tension in the lung results from oxygen diffusing from the alveoli rather than being delivered with the blood stream of the bronchial arteries. Hypoxia in the lung is therefore most frequently a result of blocked airflow through the bronchial tree into the alveoli. In the lung, the vascular system responds to hypoxia with vasoconstriction rather than vasodilation. This obvious difference between hypoxic systemic vasodilation and hypoxic pulmonary vasoconstriction has aroused intense research interest for many decades ever since it was first described in the early 20th century (7, 8). However, its molecular mechanisms have remained elusive to this date.

**Figure 1:**
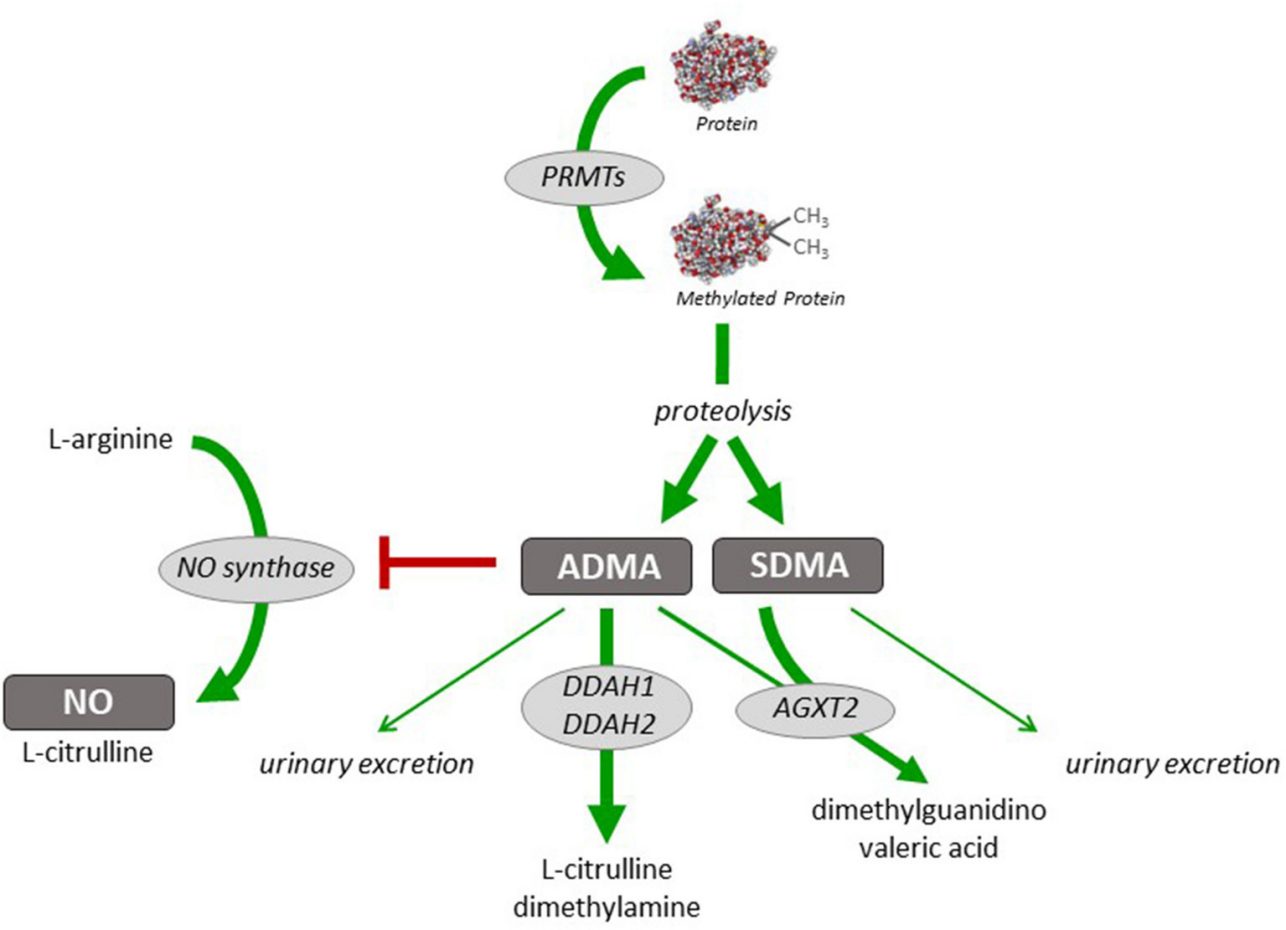
Schematic representation of pathways of dimethylarginine biosynthesis and metabolism. Dimethylarginines are formed during (di-)methylation of protein-bound L-arginine residues by a family of protein arginine N-methyltransferases (PRMTs). Free ADMA and SDMA are released during physiological hydrolytic protein turnover. Asymmetric dimethylarginine (ADMA) inhibits nitric oxide (NO) synthesis from L-arginine, whilst symmetric dimethylarginine (SDMA) does not directly interfere with NO synthase activity. ADMA is metabolically degraded to L-citrulline and dimethylamine by either of two isoforms of dimethylarginine dimethylaminohydrolase (DDAH). Both ADMA and SDMA can be cleaved by alanine glyoxylate aminotransferase-2 (AGXT2); this enzyme is the major pathway of SDMA clearance. Minor amounts of both ADMA and SDMA can also be excreted into the urine.

Nitric oxide (NO) is a critically important mediator of vasodilation under a variety of physiological and pathophysiological conditions. The generation of NO, which occurs mainly in the vascular endothelium, is regulated (a) by transcriptional and posttranscriptional mechanisms affecting the NO-producing enzyme, endothelial nitric oxide synthase (eNOS), (b) by factors regulating the enzymatic activity of eNOS, and (c) by reactive oxygen species that rapidly react—and thereby inactivate—NO once released from the endothelium. The enzymatic activity of eNOS is also regulated by the presence of methylarginines (9). Asymmetric dimethylarginine (ADMA) is a competitive inhibitor of eNOS; elevated ADMA concentration has been shown to lead to impaired NO generation and endothelial dysfunction which is reversible by L-arginine (10). Individuals with elevated circulating ADMA concentration are at increased risk of cardiovascular events and mortality (11, 12). ADMA levels are regulated through its biosynthesis, which occurs during arginine methylation of proteins by protein arginine N-methyltransferases (PRMTs) (13, 14), and through its metabolism, which is facilitated by dimethylarginine dimethylaminohydrolases (DDAH) 1 and 2 (15, 16). An alternative metabolic pathway is mediated by alanine glyoxylate aminotransferase-2 (AGXT-2) (17, 18). Dysregulation of the activity or expression of enzymes regulating ADMA concentration may thus contribute to impaired NO generation, endothelial dysfunction, vasospasm, and elevated vascular resistance, both in the systemic and pulmonary circulation (19). Figure 1 depicts the enzymatic pathways involved in the biosynthesis and degradation of ADMA.

This review aims to summarize our current understanding of the molecular mechanisms and clinical significance of hypoxic pulmonary vasoconstriction, and addresses the possible role of dysregulation of the L-arginine - dimethylarginine - NO pathway in this condition, based on recent experimental and clinical studies.

## The Physiology of Hypoxic Pulmonary Vasoconstriction

**Figure 2:**
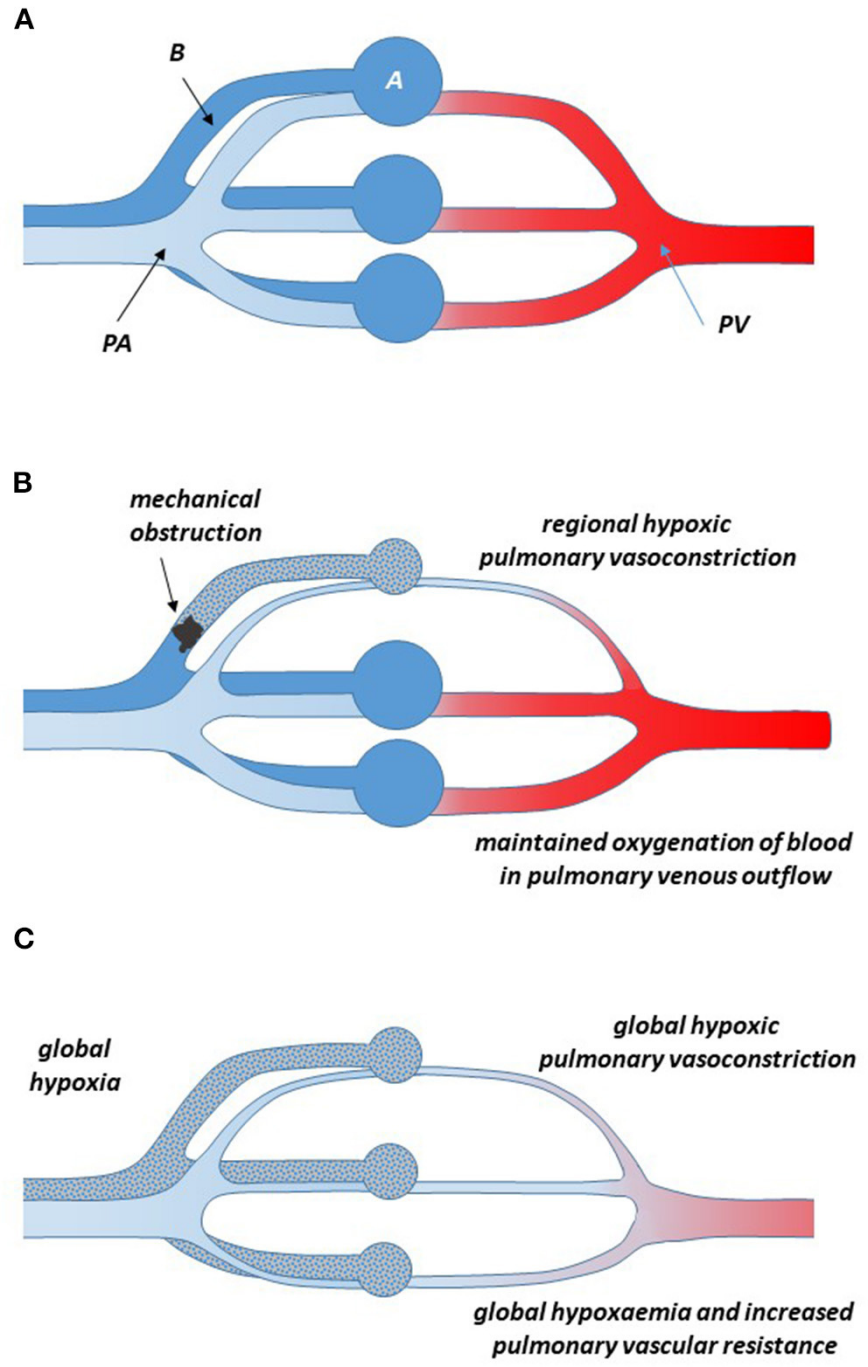
Schematic representation of the pulmonary circulation in normoxia **(A)** and when one bronchus is obstructed and the respective alveoli are hypoventilated **(B)**. During normoxia in the healthy state, deoxygenated blood from the pulmonary artery flows through the capillary bed surrounding the alveoli, where it takes up oxygen and, fully oxygenated, returns through the pulmonary vein to the left atrium of the heart. Local hypoventilation of an area of the lungs causes vasoconstriction of the pulmonary arteries in the same area; thus, less blood flows through the hypoventilated area and relatively more through other, better ventilated areas, resulting in a minimal reduction of the oxygenation status of the blood returning into the systemic circulation through the pulmonary vein (Euler-Liljestrand mechanism). **(C)** In global hypoxia, hypoxic pulmonary vasoconstriction occurs throughout the lung. This obviously does not improve the oxygenation status of the blood, but it causes a major increase in total pulmonary vascular resistance. When this situation is maintained for longer time periods, pulmonary hypertension may occur, resulting in right ventricular hypertrophy and failure.

Obviously, the lung's physiological function is to deliver fully oxygenated blood into the systemic circulation. Any regional reduction in lung ventilation—as it may occur by blocked airflow through the bronchial tree—threatens to result in suboptimal oxygenation of the blood delivered from the lung into the systemic circulation. Therefore, pulmonary vasoconstriction in a region of hypoventilation is a mechanism to redirect blood flow to better ventilated areas of the lungs, ensuring optimal oxygen supply to all tissues (Figures 2A,B).

It was the seminal work of Euler and Liljestrand in pulmonary arteries of the cat who first linked pulmonary vasoconstriction to the maintenance of full oxygenation of the blood (20). They concluded that “*[…] oxygen want and carbon dioxide accumulation have exactly the reverse local effects on the vessels of the systemic and pulmonary circulations, respectively […]. They cause a dilatation of the vessels of the working organs which need a greater blood supply than during rest, but they call forth a contraction of the lung vessels, thereby increasing the blood flow to better aerated lung areas, which leads to improved conditions for the utilization of the alveolar air*.” [quotation from Euler and Liljestrand (20)]. Ever since, this phenomenon has been known as the Euler-Liljestrand-mechanism. In 1955, Blakemore and co-workers demonstrated the existence of this same mechanism in humans. In healthy human subjects, they ventilated one lobe of the lung with physiologically oxygenated air and the other lobe with only 5% oxygen. They observed a redistribution of pulmonary blood flow toward the better oxygenated lobe of the lung (21).

## Clinical Relevance of Hypoxic Pulmonary Vasoconstriction

Physiologically, hypoxic pulmonary vasoconstriction (HPV) is a mechanism maintaining ventilation-perfusion matching and ensuring optimal oxygenation of blood. Table 1 summarizes clinical conditions in which HPV plays a pathophysiological role. Redirection of blood flow within the lung may become relevant to limit the detrimental influence of a pathogen in pneumonia, where HPV helps to divert blood flow away from regions of inflammatory infiltration toward healthy lung areas (34). However, the vasoconstrictor mechanism may become diminished in chronic pulmonary infection, and patients may experience hypoxemia in severe pneumonia (35). In bronchial asthma, bronchoconstriction may be spatially distributed in different parts of the lung; again, HPV helps to maintain ventilation-perfusion matching and minimize hypoxemia (31, 41).

**Table 1:** Clinical conditions associated with pulmonary hypoxia.

**Clinical condition**	**Role of HPV**	**Clinical significance**	**References**
**High altitude**			
High altitude pulmonary edema	Acute, extensive HPV leading to over perfusion of patent vessels with leakage of protein	Development of pulmonary edema, cyanosis, and tachycardia in unacclimatized individuals	(22)
Chronic hypobaric hypoxia (CH)	Global HPV increases pulmonary perfusion pressure	Development of pulmonary hypertension and right ventricular hypertrophy	(23)
Chronic intermittent hypobaric hypoxia (CIH)	Repeated adaptation to high altitude causes cycling between global HPV and phases of relief	Development of pulmonary hypertension and right ventricular hypertrophy	(24, 25)
Altitude training in athletes	Global hypobaric hypoxia causes HPV	HPV may impede right ventricular function and exercise performance at altitude	(26)
**Pathophysiological adaptation**			
Birth	Occurrence of HPV as local homeostatic response to focal pneumonia or atelectasis	Optimization of systemic pO_2_ without alteration of pulmonary artery pressure	(27)
Single-lung anesthesia	Reduction of blood flow to the non-ventilated lung	Facilitation of thoracic surgery, e.g., lung tumor resection	(28)
**Lung diseases**			
Sleep apnea syndrome	Intermittent apnea causes recurrent HPV and right ventricular failure	Development of pulmonary hypertension and right ventricular hypertrophy	(29, 30)
Asthma	HPV contributes to ventilation/perfusion matching in phases of acute bronchoconstriction	Maintenance of optimal oxygenation of blood	(31, 32)
COPD	HPV contributes to ventilation/perfusion matching, but is maintained chronically	Development of pulmonary hypertension	(33)
Pneumonia	Diversion of blood flow away from regions of inflammatory infiltration; in chronic pneumonia, HPV is reduced	Maintenance of optimal oxygenation of blood	(34, 35)
Interstitial lung disease	HPV is one mechanism leading to pulmonary hypertension	Deterioration of symptoms, functional capacity, and survival	(36)
Chronic thromboembolic pulmonary hypertension	HPV is aggravated by NO deficiency	Vasoconstriction and vascular remodeling trigger global pulmonary small vessel disease	(37)
Atelectasis	Diversion of blood flow away from malventilated lung area	Lessened contribution of atelectasis to right-to-left shunt and subsequent systemic hypoxaemia	(38)
ARDS	HPV is impaired in ARDS, contributing to hypoxaemia	Development of pulmonary hypertension and right ventricular failure	(39)
COVID-19	Pulmonary endotheliitis may impair HPV	Exaggerated systemic hypoxaemia and organ failure	(40)

*ARDS, acute respiratory distress syndrome; COPD, chronic obstructive lung disease; CH, chronic hypoxia; CIH, chronic intermittent hypoxia; HPV, hypoxic pulmonary vasoconstriction*.

HPV is also a mechanism keeping blood flow away from the still collapsed lungs in the fetus (32). However, after birth, focal atelectasis and pneumonia may occur. HPV helps to optimize systemic arterial oxygen pressure without altering pulmonary artery pressure (42).

### Chronification of Hypoxic Pulmonary Vasoconstriction

When ventilation obstacles become chronic like in chronic obstructive lung disease, hypoxic pulmonary vasoconstriction often persists. Acting together with inflammatory and adaptative processes that stipulate remodeling of and fibrosis in the pulmonary vasculature (43), this may lead to persistently elevated pulmonary vascular resistance and structural changes in the pulmonary vascular walls during the progression of the disease and be a cause of pulmonary hypertension, right ventricular hypertrophy, and—finally—failure (33, 44). In chronic thromboembolic pulmonary hypertension (CTEPH, also classified as group IV of the WHO classification of pulmonary hypertension), thrombotic occlusion of a segmental pulmonary artery *per se* increases total pulmonary vascular resistance; However, secondary mechanisms may be triggered in the non-occluded pulmonary vessels that cause vascular remodeling and lead to a progressive further increase in total pulmonary vascular resistance (45, 46).

### Global Pulmonary Hypoxia

Another cause of pathological consequences of HPV is exposure to global pulmonary hypoxia (Figure 2C). This may occur at high altitude, when hypoxia results from the low ambient pressure (hypobaric hypoxia). Acute exposure of non-acclimatized individuals to high altitude, as it can be seen in unexperienced climbers and tourists engaging in mountaineering activities, can lead to high-altitude pulmonary oedema (22). This oedema results from global but heterogeneous HPV with increased pulmonary perfusion pressure acting on the capillary bed, which becomes leaky to protein (47). High altitude pulmonary oedema can be resolved by returning to sea level (22). Residents of high altitude of different ethnic origins show different levels of adaptation to the consequences of chronic global pulmonary hypoxia. Indians native to the Andean highlands at 3,500–4,000 m have a high prevalence of hypoxic pulmonary hypertension (23), whilst inhabitants of the Tibetan plateau living at altitudes of ≥ 3,500 m rarely develop polycythaemia and pulmonary hypertension (48). One major factor contributing to altitude adaptation in Tibetans was reported to be accumulation of genetic polymorphisms in EGLN1, the gene encoding for HIF-2α (49–51). This is in accordance with the important role of HIF-2α in hypoxia-induced upregulation of erythropoietin expression (52).

The main desired effects of high altitude training also depend on hypoxia-inducible factor-2α (HIF2α)-mediated regulation of gene expression, e.g., transcriptional upregulation of erythropoiesis and subsequent improvement in oxygen transport capacity of the blood. However, the combined decreases in arterial oxygen saturation and cardiac output at altitude may limit aerobic exercise capacity, which can be resolved when lowering pulmonary arterial pressure, e.g., by treatment with an ET-1 antagonist (53), but not by acetazolamide treatment (54).

Diminished HPV may be a common mechanism of adaptation to life at high altitude: Cattle native to lowlands exhibit marked hypoxic pulmonary vasoconstriction when exposed to high altitude, resulting in an incidence of about 20% of pulmonary hypertension, pulmonary oedema, and right ventricular failure (55), a condition named brisket disease after the resulting oedema in the cows' necks (56). Interestingly, neonatal calves chronically exposed to high altitude progressively loose the vasodilator response of pulmonary arteries to acetylcholine, a well-characterized stimulus of endothelial NO release (57). This finding points to diminished NO-mediated pulmonary arterial vasodilation as a possible contributor to HPV. By contrast, yaks native to the high altitude of the Himalayan region exhibit diminished HPV and maintain low pulmonary arterial pressure (58). A recent study showed that yaks differ from cattle by lower circulating levels of ADMA and higher protein expression and activity of DDAH, the enzyme inactivating ADMA (59), supporting a role for modulation of the NO pathway in adaptation of the pulmonary circulation to high altitude.

A clinical condition that has been more recently defined is called chronic intermittent hypobaric hypoxia. Workers in mines of the Andean plateau at altitudes above 3,500 m, frontier officials, and other individuals may be exposed to working shifts alternating between several days at high altitude, followed by a few days of rest at sea level (60, 61). This leads to frequent cycling of affected individuals between the acute adaptation to hypoxia at high altitude and relief. In consequence, changes to the pulmonary circulation may occur that are very similar and may be as severe as in chronic hypobaric hypoxia (24, 62). The prevalence of elevated mean pulmonary arterial pressure (mPAP) with mPAP ≥ 25 mm Hg was reported to be as high as 26% and the prevalence of high altitude pulmonary hypertension [the threshold of which has been defined at mPAP ≥ 30 mm Hg (63)] was about 9% in chronic intermittent hypobaric hypoxia (24). Based on a meta-analysis of multiple large cohorts, systolic pulmonary arterial pressure (sPAP) at sea level was calculated to be (median [95% CI]) 18.4 [17.1–19.7] mm Hg, whilst sPAP at high altitude was 25.3 [24.0–26.7] mm Hg (64). As the threshold of mPAP for the definition of pulmonary arterial hypertension in lowlanders has recently been reduced to mPAP ≥ 20 mm Hg (65), an updated, evidence-based definition of pulmonary arterial hypertension at high altitude appears urgently needed (66).

Pulmonary hypertension is also one pathological consequence of chronic intermittent hypoxia in obstructive sleep apnoea syndrome (OSAS); increased pulmonary arterial pressure may occur during sleep, but also during waking hours (29). Whilst clinically relevant pulmonary hypertension is rare in pure OSAS, it may occur much more frequently in the so-called overlap syndrome, i.e., the combined occurrence of OSAS and chronic obstructive pulmonary disease (COPD) (30). Although there still remain gaps in our understanding of the pathophysiology of this relationship (67), one relevant observation helping us to understand the association of OSAS with vascular disease in both, the pulmonary and systemic circulation, is the presence of endothelial dysfunction, i.e., the inability of the vascular endothelium to generate physiological amounts of NO as required to maintain vasodilator tone (68).

Recent interest has focussed on the role of pulmonary vascular damage and endothelial dysfunction in COVID-19 pneumonia and ensuing hypoxaemia and organ failure (69, 70). We have reported that high ADMA and SDMA serum levels are superior biomarkers to predict COVID-19-associated in-hospital mortality (71), suggesting that NO deficiency may aggravate pulmonary and systemic vascular dysfunction in this disease. Accordingly, several small trials investigated the effects of inhaled NO (72, 73) or the phosphodiesterase V inhibitor sildenafil on COVID-19-associated hypoxaemia and outcome (74). However, the reported results of these studies have so far been inconclusive.

## Mechanisms of Hypoxic Pulmonary Vasoconstriction

The best known transcriptional regulators of the physiological responses are the hypoxia-inducible factors (HIF). HIF-1α is activated acutely upon oxygen deficiency, whilst HIF-2α mediates the sustained responses to prolonged hypoxia (75). By this mechanism, hypoxia elicits a systemic hemodynamic response via activation of the carotid chemokine receptors and systemic humoral mechanisms. In addition, hypoxia also acts locally on the pulmonary vessels, thereby modulating the relation between pulmonary blood flow and alveolar ventilation. Although HIF-1 target genes have been shown to be involved in the pulmonary arterial response to hypoxia (76), the cellular crosstalk in the hypoxic lungs appears to be more complex, and the exact molecular and cellular nature of this local mechanism of HPV has remained elusive so far. A number of determinants can be defined, however, that are prerequisites of a locally functioning physiological mechanism:

- There must be an oxygen sensor at the level or in the immediate adjacency of the pulmonary alveoli and pulmonary blood vessels.
- There must be a locally functioning vasoconstrictor mechanism activated and / or vasodilator mechanism diminished by hypoxic signaling. This mechanism must be rapidly activated, reversible in nature, and evocable by mild hypoxia.

There are three major cell types in the lung, of which each may be responsible for initiating HPV: endothelial cells and vascular smooth muscle cells of the pulmonary arterioles, and alveolar epithelial cells lining the bronchioli and alveoli. The endothelial cells form the physiological barrier between the circulating blood and the adjacent vascular tissue, they are the major source of effectors influencing the vasoconstrictor and vasodilator properties of blood vessels. As such, they are predisposed to interlace between changes in tissue oxygen content and vascular tone by generating vasoactive mediators (see below). The vascular smooth muscle cell is less easily capable of sensing the blood oxygen content due to its more distant spatial localization. However, a hypothetical oxygen sensor located in the vascular smooth muscle cell itself could directly modulate the cell's contractile properties. The alveolar epithelial cells, on their turn, are the primary cells exposed to low oxygen content in the breathing air, and therefore predisposed to act as sensor cells. Thus, the complexity of this intercellular cross-talk may at least partly explain that the exact molecular mechanism of HPV has not yet been unraveled. Finally, different cell types or signaling mechanisms may be involved in mediating the early and late phases of HPV.

### Oxygen Sensing

One of the most extensively studied sites of oxygen sensing is the carotid body, which regulates major neuroendocrine responses to hypoxemia. Carotid body glomus cells respond to hypoxemia by inhibition of K^+^ channels, leading to membrane depolarization, calcium influx via voltage-gated Ca^2+^ channels, and neuroendocrine secretion (77, 78). In the pulmonary circulation, the cellular and molecular identity of the oxygen sensor has remained much less clear. Experiments demonstrating that redox agents and certain inhibitors of complexes I and III of the mitochondrial electron transport chain cause vasoconstriction in the pulmonary vascular bed, but vasodilation in the fetal ductus arteriosus (79)—mimicking the differential responses to hypoxia in these two vascular beds—suggest that redox mechanisms may be involved. Thus, research to identify the pulmonary oxygen sensor has focused on NADPH oxidases and on the mitochondrial respiratory chain (78), and models aiming to explain HPV based on mitochondrial oxygen sensing have been proposed (80–82). In line with this, knockdown of NADH dehydrogenase ubiquinone iron-sulfur protein-2 (Ndufs-2) within the mitochondrial complex I significantly decreased hypoxic vasoconstriction in pulmonary artery smooth muscle cells (83). Another source of oxygen-derived radicals during hypoxia and ischemia episodes is accumulation of succinate, an intermediate metabolite in the mitochondrial citric acid cycle (84). Accumulation of succinate stimulates mitochondrial production of reactive oxygen species by reversing electron transport at mitochondrial complex I (85). Through this mechanism, succinate overload in hypoxia is known to activate HIF-1α (86). During normoxia, the HIF-1α protein is hydroxylated by prolyl hydroxylases that are absolutely dependent on the presence of oxygen. Hydroxylation enables binding of HIFs to the ubiquitin proteasome system and subsequent degradation; inhibition of this degradation pathway in hypoxia activates HIF-mediated gene transcription (75, 76).

Recent studies also suggest that pulmonary and systemic arteries share the same oxygen sensing mechanism within mitochondria, whilst differences in downstream signaling of reactive oxygen species released from hypoxic mitochondria cause site-specific vascular responses (87). As the three major cell types present in the lung have all been shown to be responsive to hypoxia (81, 88, 89), the cellular location of the oxygen sensor has remained controversial.

### Signal Transduction and Effector Mechanisms: The Vascular Smooth Muscle Cell

HPV is brought about by a contractile response of the pulmonary vascular smooth muscle cells (VSMC). Smooth muscle cell contraction is highly dependent on elevated cytosolic calcium concentration; therefore, the effector mechanisms responsible for HPV likely involve modulation of VSMC calcium handling. Sarcoplasmic calcium channels, voltage-dependent potassium channels, transient receptor potential channels, and L-type calcium channels are the main regulators of cytosolic calcium (90). The coordinated response of these ion channels is influenced by protein kinases and reactive oxygen species (ROS). The Ca^2+^ influx directly triggers a conformational change of the myosin light chain, thereby facilitating interaction with actin filaments and contraction. Several studies have provided evidence for an involvement of ion channels in HPV: For example, inhibition of voltage-dependent potassium channels caused vasoconstriction in the isolated perfused rat lung (91). Furthermore, inhibition of L-type calcium channels diminished whereas activation of these channels enhanced the vasoconstrictor response to hypoxia (92, 93). However, the modulation of vascular tone by these channels does not differ between systemic and pulmonary arteries. Therefore, this mechanism cannot explain the heterogeneous response to hypoxia (vasoconstriction *vs*. vasodilation) in pulmonary and systemic arteries, respectively.

### Signal Transduction and Effector Mechanisms: The Vascular Endothelial Cell

**Figure 3:**
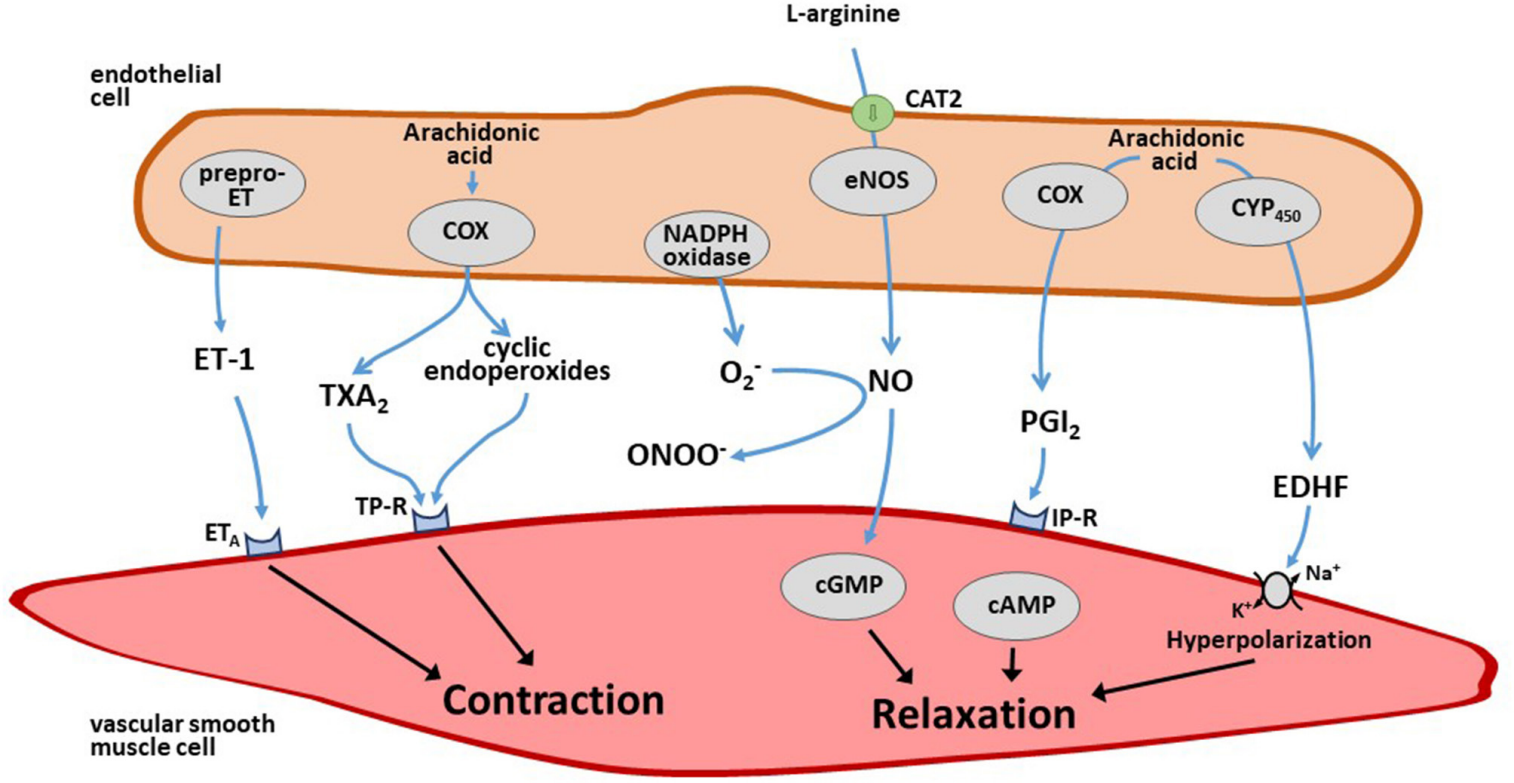
Schematic overview of endothelium-derived vasoconstrictor and vasodilator mediators. The endothelium produces several vasoconstrictor mediators like endothelin-1 (ET-1) and thromboxane (TX) A_2_ as well as vasodilator mediators like nitric oxide (NO), prostacyclin (PGI_2_), and endothelium-derived hyperpolarizing factor (EDHF) that diffuse to the adjacent smooth muscle cells that effect changes in vascular tone upon this stimulation. For further details see text.

Endothelium-derived vasoactive mediators are major regulators of vascular tone in the systemic circulation. The endothelium-dependent vasoconstrictor substances include the peptide endothelin-1 (ET-1) (94), superoxide anions (95), and arachidonic acid-derived endoperoxides and/or thromboxane A_2_ (96). The endothelium-derived relaxing factors include NO, prostacyclin, and endothelium-derived hyperpolarizing factor (EDHF) (97). Both endothelial vasoconstrictor and vasodilator mediators are finely tuned to maintain the homeostasis of local blood flow and its adaptation to varying needs of oxygen and nutrient demand (Figure 3). Less information is available about the role of endothelium-derived mediators in the regulation of pulmonary vascular tone.

ET-1 is the most potent vasoconstrictor peptide released by endothelial cells (94). Human ET-1 is synthesized as a 212-amino acid peptide (prepro-ET-1); it exerts a long-lasting vasoconstrictor effect by activating ET_A_ receptors (98). By contrast, binding of ET-1 to ET_B_ receptors, which are located on the endothelial cell membrane, causes vasodilation and anti-mitogenic effects through the release of NO and/or prostacyclin (PGI_2_) (99, 100). The lung is an important site of ET-1 production, with ET-1 mRNA being five times more abundant in the lung than in other organs (101). Lowering oxygen levels in cultured endothelial cells rapidly increases the mRNA expression of prepro-ET-1 (102). This effect persists for at least 48 h when hypoxia is maintained, and it is reversible after increasing oxygen tension to normal ambient pressure. These experimental findings are in line with *in vivo* observations from animal studies (103–105), and with the observation that circulating ET-1 is elevated in COPD patients with chronic hypoxia (106). However, the endothelin receptor antagonist bosentan had variable effects on HPV in animal models and clinical studies (107–109). This may be due to the fact that bosentan is a dual blocker of both ET_A_ and ET_B_ receptors. Hypoxia enhances the expression of ET_A_ and ET_B_ receptors in the lung, but there is evidence for a predominant upregulation of ET_B_ receptors. Thus, under hypoxic conditions, the effect of bosentan in the pulmonary circulation may be dominated by blocking ET_B_-mediated vasodilation (110).

Arachidonic acid metabolites are released from endothelial cells upon stimulation with acetylcholine, serotonin, adenosine diphosphate (ADP), and other substances. Based on the expression of cyclooxygenase and the spectrum of prostaglandin synthases in a specific cell type, either the vasodilator metabolites prostacyclin and PGE_2_, or the vasoconstrictor endoperoxides and thromboxane A_2_ may be released. For example, stimulation of isolated aortic rings from Wistar rats with acetylcholine results in endothelium-dependent vasodilation, whereas aortic rings from spontaneously hypertensive rats (SHR) respond with vasoconstriction (111). Aortic vasoconstriction in SHR is enhanced when endothelial NO production is blocked, whilst vasodilation is unmasked when cyclooxygenase activity is blocked (112). During chronic hypoxia, mouse pulmonary arteries release less prostacyclin and more 8-iso-prostaglandin F_2α_ [a lipid peroxide product derived from non-enzymatic oxidation of arachidonic acid by superoxide anion (113)]. Cyclooxygenase-2 is upregulated, and endothelium-dependent relaxation in normoxia is shifted to an endothelium-independent, thromboxane receptor-dependent contraction (114).

NO is the major endothelial vasodilator mediator in the systemic and in the pulmonary circulation. In most arterial beds, it is only under pathophysiological conditions when NO signaling is impaired or under experimental conditions when NO production is pharmacologically or genetically inhibited that a significant role can be determined for other endothelial mediators. During the recent years, our research has focused on the regulation of the NO pathway by endogenous, methylated analogs of L-arginine, the physiological precursor of NO (115, 116). Evidence has accumulated that dysregulation of the NO pathway by ADMA may be involved in HPV and pulmonary hypertension (117).

### Signal Transduction and Effector Mechanisms: The Alveolar Epithelial Cell

Alveolar epithelial cells are the cell type most directly exposed to decreased oxygen content in the inspired air. Type II alveolar epithelial cells make up about two thirds of the alveolar epithelial surface in the normal human lung; they play an important role in surfactant production and recycling (118). Early experiments had shown that in the isolated perfused cat lung, ventilation with low oxygen gas increased, but perfusion with partially deoxygenated blood did not increase pulmonary vascular resistance, suggesting that oxygen content in the inspired air, but not hypoxemia in the pulmonary blood vessels stipulates HPV (119). More recent experiments showed differential effects of hypoxia on human alveolar epithelial cells and human pulmonary microvascular endothelial cells, respectively, with the alveolar epithelial cells displaying a more sensitive response to hypoxia (120). Others revealed that acute changes in inspired oxygen tension are sensed by large conductance calcium-activated potassium channels of human alveolar epithelial cells (121), causing membrane hyperpolarization. Beyond that, alveolar epithelial cells are capable of secreting paracrine mediators which may influence the function of adjacent endothelial and vascular smooth muscle cells; amongst such mediators, NO derived from inducible NOS in type II alveolar epithelial cells (122), interleukin-33, and the receptor for advanced glycation end products (RAGE) have been identified [for review, cf. (89)]. Thus, alveolar epithelial cells may be involved in sensing hypoxia and mediating this signal to vascular endothelial and smooth muscle cells, thereby contributing to pulmonary vascular contraction and remodeling in hypoxia (123).

## Dysregulation of the Endothelial NO Pathway in the Hypoxic Pulmonary Circulation

Acute and chronic hypobaric hypoxia at high altitude result in endothelial dysfunction, a situation defined by impaired endothelium-dependent, NO-mediated vasodilation in response to brief phases of ischemia in the forearm or in response to local infusion of acetylcholine. Endothelium-dependent vasodilation is acutely impaired in lowlanders after arrival to high altitude hypoxia (124) as well as in Tibetan inhabitants of the Himalaya region, despite the good genetic adaptation of this population to chronic hypobaric hypoxia (125). Inhabitants of the Andean high altitude region also show distinct endothelial dysfunction, which is more pronounced in individuals with cardiovascular risk factors or overt cardiovascular disease than in controls (126).

The underlying mechanisms leading to dysfunction of the NO pathway have been extensively studied and are considered to be multifactorial. Changes in eNOS gene expression, reduced eNOS catalytic activity, altered L-arginine metabolism, and increased NO consumption by reaction with superoxide anion may all contribute to a lack of bioactive NO.

There is evidence of markedly decreased eNOS gene expression in the endothelium of patients with pulmonary hypertension (127). However, subsequent studies found pulmonary expression of eNOS unchanged in pulmonary hypertension (128), and some studies even reported increased expression of eNOS and/or the inducible isoform of NOS (129). Thus, NOS gene expression does not always correspond to NO production, as NOS activity may be influenced by several factors relevant to pulmonary hypoxia.

Endothelial NOS needs a variety of co-factors to function normally [reviewed in Förstermann and Sessa (130) and Moncada and Higgs (131)]. When the endothelial cell is depleted of co-factors, eNOS becomes “uncoupled,” i.e., its catalytic activity is driven toward the generation of superoxide anions (130). Specifically, oxidation of the essential eNOS co-factor tetrahydrobiopterin has been shown to cause uncoupling of eNOS activity and endothelial dysfunction.

Another cause of diminished eNOS activity may be the presence of endogenous NOS inhibitors. Table 2 summarizes experimental evidence from animal models for a link between dimethylarginine metabolism, hypoxia, and pulmonary arterial hypertension. ADMA is produced during the post-translational methylation of arginine residues within specific proteins (13, 144). When methylated proteins are cleaved, ADMA is released instead of L-arginine. ADMA competes with L-arginine for binding to the NOS catalytic site and thus competitively inhibits NOS activity. Another dimethylarginine, symmetric dimethylarginine (SDMA), is unable to directly interfere with NOS activity, but like ADMA, it may inhibit CAT-2, the cellular uptake transporter for L-arginine (145, 146). We have recently reviewed in detail the transcriptional and post-translational mechanisms of regulation of dimethylarginine metabolism (9). Dimethylation of proteins occurs as a process of posttranslational protein modification and leads to increased hydrophobicity of the respective protein moieties. This process is ubiquitously present in all tissues investigated so far, although the specific types of protein arginine N-methyltransferases (PRMT) may vary in a tissue-specific manner. Amongst highly dimethylated proteins are heterogeneous nuclear ribonucleoproteins. Histone proteins are activated by asymmetric dimethylation and repressed by symmetric dimethylation, this affects their regulatory roles in gene expression (147, 148). Myelin basic protein is a neuronal protein that is known to be highly symmetrically dimethylated (149), a fact that may explain why high SDMA concentrations can be found in cerebral ischemic stroke (150, 151). Physiological turnover of proteins releases either ADMA or SDMA, depending on the type of methylation of the degraded protein. Although several PRMT enzymes are expressed in the lungs, it is not known whether asymmetric or symmetric demethylation plays a functional role in the lungs or in the vascular system.

**Table 2:** Experimental models linking derangement of the ADMA/DDAH pathway with pulmonary hypoxia and pulmonary vascular dysfunction.

**Experimental condition**	**Study design**	**Functional consequence**	**References**
1 week of HX in rats	Exposure of adult male rats to 1 week of HX (10% O_2_)	1.9-fold ↑ in eNOS protein and 37% ↓ in DDAH1 protein in lungs of HX rats; pulmonary ADMA ↑ by 2.3-fold, DDAH activity ↓ by 37% and NO ↓ by 22%, respectively	(132)
Newborn piglets during normal postnatal development and in PPHN	Analysis of DDAH1 and DDAH2 protein and of DDAH activity in lungs	DDAH1 protein remained unchanged, whilst DDAH2 protein was ↑ after birth; in PPHN DDAH2 protein and DDAH activity were ↓ but DDAH1 protein unchanged	(133)
CH in mice	3 weeks of hypoxia (10% O_2_)	In CH: PRMT2 ↑ in alveolar type II cells; ADMA ↑ and ADMA/L-arginine ratio ↑	(134)
HX exposure with and without hypoxic conditioning in mice	Acute HX exposure after hypoxic (HC) or sham conditioning (SC), with or without i.p. injection of ADMA	ADMA increased HX survival time in HC and in SC mice; the effect was mediated by regulation of eNOS activity	(135)
DDAH-1^+/−^ mice	DDAH-1 expression, DDAH-2 expression, ADMA	Hypertension, endothelial dysfunction, right ventricular pressure	(136)
Allergically inflamed mouse lungs	Ovalbumin sensitization, ovalbumin + L-arginine treatment, control mice	PRMT2 ↑ and DDAH2 ↓ in ovalbumin-treated mice, along with ↑ ADMA and ↑ nitrotyrosine; Reversal with oral L-arginine treatment	(137)
Acute and chronic hypoxia in DDAH1-transgenic and WT mice	Acute (10 min) and sustained HX (3 h) in isolated perfused mouse lungs; chronic HX (4 weeks);	No change in acute HPV in DDAH1 transgenic mice vs. WT; decreased sustained HPV in DDAH1 transgenic mice vs. WT; no difference in CH-induced PAH	(138)
Peritoneal macrophages from macrophage-specific DDAH2 k.o. and WT mice	Exposure of macrophages to HX (3% O_2_) followed by reoxygenation	NO_x_ production increased in WT monocytes after HX; DDAH2 protein increased by 4.5-fold and ADMA decreased by 24% after HX; DDAH2 k.o. abolished the HX-induced changes in NO_x_ and ADMA	(139)
Chronic intermittent normobaric hypoxia	Diabetic and non-diabetic mice subjected to chronic intermittent normobaric hypoxia or control for 8 weeks	↓ endothelium-dependent vasodilation and ↑ ADMA in hypoxic mice vs. controls	(140)
CIH in rats	Exposure of Wistar rats to CIH, CH, or NX for 30 days	↑ RVH in CIH and CH vs. NX; lung eNOS mRNA ↑ in HX groups, but NOS activity unchanged, ADMA ↑. DDAH activity ↓ only in CH	(141)
CH in DDAH1-transgenic and WT mice	Exposure of WT and DDAH1-transgenic mice to HX (10% O_2_) for 2 weeks	↑ RVSP and ↑ RVH as well as ↑ DDAH1 protein in lungs of hypoxic mice; attenuation of ↑ RVSP and ↑ RVH in DDAH1-transgenic mice	(142)
CH in DDAH1 k.o. and WT mice	Exposure of DDAH1 k.o. and WT mice to 3 weeks of CH	ADMA ↑ in WT lungs during HX; DDAH1 mRNA and protein ↓ in WT lungs; DDAH2 protein ↑ in DDAH1 k.o. lungs during HX; no difference in RVH and RVSP between genotypes	(143)

*ADMA, asymmetric dimethylarginine; CH, chronic hypoxia; CIH, chronic intermittent hypoxia; DDAH, dimethylarginine dimethylaminohydrolase; eNOS, endothelial nitric oxide synthase; HC, hypoxic conditioning; HPV, hypoxic pulmonary vasoconstriction; HX, hypoxia; i.p., intraperitoneal; NX, normoxia; PRMT, protein arginine N-methyltransferase; RVH, right ventricular hypertrophy; RVSP, right ventricular systolic pressure; SC, sham conditioning; WT, wild-type*.

ADMA is mainly degraded by the enzyme dimethylarginine dimethylaminohydrolase (DDAH), which exists in two isoforms. DDAH-1 has been described as the major isoform in the kidneys and liver, whilst DDAH-2 is expressed mainly in vascular tissues (16, 152). Derangement of DDAH, either genetically induced in knockout mouse models, pharmacologically caused by DDAH-inhibitory compounds, or biochemically caused by high glucose or oxidative stress, leads to elevated ADMA that impairs NO generation by eNOS and results, amongst other effects, in elevated pulmonary arterial pressure (136).

## Evidence for Dysregulation of the Dimethylarginine Pathway in Pulmonary Hypoxia and Pulmonary Arterial Hypertension

In patients with different pulmonary diseases, ADMA levels are higher than in healthy controls (Table 3). Specifically, elevated ADMA has been reported in patients with obstructive sleep apnoea syndrome (OSAS) and in those with chronic obstructive lung disease (COPD). Both conditions are associated with hypoxemia, the development of elevated pulmonary artery pressure, pulmonary arterial hypertension, and right heart failure, as well as a high risk of systemic cardiovascular disease (183, 184). Multiple small cross-sectional studies reported higher plasma or serum ADMA in COPD than healthy controls; in addition, some studies reported an inverse correlation between ADMA and FEV_1_ or COPD severity grade (167, 172), or significantly higher ADMA in acutely exacerbated than in stable COPD (170, 173). High ADMA was associated with intima-media thickness in the brachial artery of COPD patients (169) and inversely associated with serum NO metabolites in another study (170). Lastly, ADMA and SDMA had prognostic relevance in a prospective study with 150 patients with acutely exacerbated COPD; the highest quartiles of ADMA and SDMA were significantly associated with all-cause mortality after 6 years of follow-up (mortality rate, 54%) (170).

**Table 3:** Clinical conditions of pulmonary hypoxia in which derangement of the ADMA / DDAH pathway was described.

**Clinical condition**	**Study design**	**Functional consequence**	**References**
**High altitude**			
Chronic-intermittent hypobaric hypoxia	72 healthy Chilean lowlanders exposed to CIH during 3 months; 16 Andean highlander natives	ADMA ↑ by 80 % in CIH; no change in SDMA in CIH; highest ADMA in highland natives	(153)
Chronic-intermittent hypobaric hypoxia	100 healthy Chilean lowlanders exposed to CIH during 6 months; echocardiography at 6 months	ADMA ↑ in CIH; SDMA ↓ in CIH; individuals with highest ADMA had highest risk of HAPH	(60)
Chronic intermittent hypobaric hypoxia	120 Chilean mining workers after exposure to CIH for a mean 14 ± 0.5 years	ADMA, but not SDMA, ↑ as compared to reference levels; higher ADMA in workers with HAPH (mPAP > 30 mm Hg) than in those without	(24)
High altitude pulmonary oedema	200 HAPE patients, 200 HAPE-free altitude sojourners, and 450 healthy highlanders	ADMA significantly ↑ in HAPE-patients and in highlanders than in HAPE-free sojourners	(154)
Acute hypobaric hypoxia (hypobaric chamber)	12 healthy humans during a 24 h stay in a hypobaric chamber	*N* = 5 developed AMS, high mPAP, and decreased ADMA; *N* = 4 had mild AMS, mildly elevated mPAP, and elevated ADMA	(155)
**Obstructive sleep apnea syndrome**			
Obstructive sleep apnea syndrome	188 OSAS patients, 520 controls	No difference in ADMA between OSAS and controls	(156)
Obesity	518 obese individuals; 242 OSAS patients, 276 non-OSAS individuals	ADMA and SDMA ↑ with increasing AHI	(157)
Obstructive sleep apnea syndrome	95 patients with suspected OSAS undergoing polysomnography	Significant inverse linear correlation between AHI and flow-mediated vasodilation in the forearm; ADMA significantly ↓ after 3 months of CPAP therapy in 63 OSAS patients with AHI>20	(158)
Obstructive sleep apnea syndrome	40 OSAS patients 20 healthy controls	ADMA ↑ in OSAS vs. controls	(159)
Obstructive sleep apnea syndrome	13 patients with severe OSAS, 13 patients with mild-to-moderate OSAS, 12 controls	ADMA not significantly higher in severe or mild-to-moderate OSAS than in controls; ADMA significantly correlated to arousal index	(160)
Obstructive sleep apnea syndrome	OSAS patients with (*N* = 23) or without (*N* = 18) concomitant CV risk factors, 23 healthy controls	ADMA ↑ in OSAS, but not related to the presence of CV risk factors	(161)
Obstructive sleep apnea syndrome	34 OSAS patients, 15 healthy controls	ADMA ↑ and NO metabolite levels ↓ in OSAS	(162)
Children with OSAS	26 children with OSAS, 8 healthy controls	No significant difference in ADMA between OSAS and control children	(163)
Obstructive sleep apnea syndrome	10 male OSAS patients before and after CPCP therapy	Significant improvement in flow-mediated vasodilation after CPAP therapy, concomitant with ↓ ADMA	(164)
**Chronic obstructive lung disease**			
COPD	29 stable COPD, 35 exacerbated COPD, 15 control smokers	Serum L-arginine/ADMA ratio ↓ in stable and exacerbated COPD; serum SDMA ↑ in COPD and decreased after systemic steroid treatment	(165)
COPD	COPD patients with or without PAH (sPAP > 35 mm Hg), healthy controls	ADMA ↑ in COPD with PAH vs. both other groups	(166)
COPD	42 patients with mild to very severe COPD, with or without PAH (sPAP > 36 mm Hg)	ADMA and SDMA ↑ with decreasing FEV_1_, but SDMA ↓again with very low FEV_1_; ADMA and SDMA slightly, but not significantly higher in COPD patients with PAH	(167)
COPD	74 COPD patients	Significant correlation of ADMA with airway resistance in patients with poorly controlled airway obstruction; ADMA significantly associated with airway resistance in multiple linear regression (*R* = 0.42 [0.06–0.77])	(168)
Stable COPD	60 patients with stable COPD, 20 smoking and 20 non-smoking healthy controls	Brachial artery intima-media thickness (IMT) ↑ in COPD than in controls; significant correlation of IMT with ADMA	(169)
Exacerbated COPD	150 patients with acute exacerbation of COPD; 6 years of prospective follow-up for total mortality	ADMA and SDMA ↑ in more severe pneumonia and with higher SOFA Score; highest quartiles of ADMA and SDMA significantly associated with all-cause mortality (54%) after 6 years	(170)
Elderly patients with stable COPD	41 COPD patients, 35 elderly controls	Bronchial obstruction (FEV_1_) associated with arterial stiffness and brachial artery flow-mediated vasodilation; no correlation with ADMA	(171)
COPD	58 COPD patients, 30 healthy controls	ADMA ↑ in COPD, whilst serum NOx ↓ in COPD—inverse correlation between both parameters; ADMA inversely correlated with FEV_1_, ADMA ↑ with progression of COPD stage	(172)
Stable and exacerbated COPD	32 patients with stable COPD, 12 patients with acute exacerbation of COPD, 30 healthy controls	ADMA and SDMA ↑ in COPD than controls; ADMA and SDMA ↑ in exacerbated vs. stable COPD	(173)
Mild to moderate COPD	43 COPD patients, 43 matched controls	Non-significant increase in ADMA in mild and moderate COPD; ADMA/arginine ratio associated with COPD severity	(174)
COPD	10 COPD patients	Sputum ADMA correlates with sputum L-ornithine and L-citrulline	(175)
**Overlap syndrome**			
COPD patients, OSAS patients, and patients with overlap syndrome (OS)	26 patients with COPD, 25 with OSAS, and 24 with OS	ADMA ↑ in COPD vs. OSAS or OS; no change in ADMA after 30 days of CPAP treatment in OSAS and OS patients	(176)
COPD patients, OSAS patients, and patients with overlap syndrome (OS)	25 patients each with COPD, OSAS, or OS	ADMA ↑ in COPD vs. OSAS or overlap syndrome; no change in ADMA after 4 weeks of CPAP treatment in OS	(177)
**Pulmonary arterial hypertension**			
Idiopathic PAH	Patients with IPAH, healthy controls	ADMA ↑ in IPAH vs. healthy controls; significant association of ADMA with right ventricular function and with mortality	(178)
PAH in systemic sclerosis	66 European patients with systemic sclerosis (24 with PAH, 42 without PAH), 30 age-matched healthy controls	ADMA ↑ in systemic sclerosis with PAH, not in systemic sclerosis without PAH	(179)
PAH in connective tissue disease	88 Chinese patients with connective tissue diseases (43 with PAH, 45 without PAH), and 40 healthy controls	ADMA ↑ in connective tissue diseases with PAH, not in connective tissue diseases without PAH	(180)
HIV-associated PAH	214 HIV patients, of whom 85 underwent right heart catheterization for suspected PAH	ADMA ↑ in HIV patients with PAH than in those without; mPAP 14.2% higher per each 0.1 μmol/L increase in ADMA	(181)
CTEPH	135 CTEPH patients, 40 healthy controls	ADMA ↑ in CTEPH patients than in controls	(182)
**COVID-19**			
Patients hospitalized with severe COVID-19	31 patients hospitalized with severe COVID-19	ADMA and SDMA ↑ in COVID-19 non-survivors than in survivors; ADMA and SDMA were best predictors of in-hospital mortality of COVID-19 patients	(71)

*AMS, acute mountain sickness; CIH, chronic intermittent hypoxia; COPD, chronic obstructive lung disease; CTEPH, chronic thromboembolic pulmonary hypertension; HAPE, high altitude pulmonary edema; HAPH, high altitude pulmonary hypertension; HIV, human immunodeficiency virus; iPAH, idiopathic pulmonary arterial hypertension; mPAP, mean pulmonary arterial pressure; OSAS, obstructive sleep apnea syndrome; PAH, pulmonary arterial hypertension; sPAP, systolic pulmonary arterial pressure*.

Data on plasma or serum ADMA concentrations are more controversial in OSAS. Some case-control studies reported higher ADMA concentration in OSAS (157, 159, 161), along with lower NO metabolite levels (162) or impaired endothelium-dependent vasodilation (185). However, other investigators were unable to reproduce these findings (156, 177). Interpretation of these studies is hampered by methodological flaws in some studies, by lack of healthy controls in others, and by differences and—in some studies—uncertainties about analytical methods utilized for ADMA quantification.

Additionally, elevated ADMA has been measured in several types of pulmonary arterial hypertension (179, 181, 182). A prospective study reported that elevated ADMA is associated with impaired long-term survival of patients with primary pulmonary arterial hypertension (178), a finding in line with the reported role of ADMA as a marker of long-term cardiovascular events and mortality in the general population (11, 186, 187).

We and others have studied the effects of chronic hypobaric hypoxia and chronic intermittent hypobaric hypoxia on the regulation of the NO/ADMA pathway in a number of experimental models and clinical cohorts. Rats that were exposed to chronic hypobaric hypoxia for 30 days developed right ventricular hypertrophy, diminished DDAH activity, and elevated circulating ADMA levels (141). Despite upregulated eNOS mRNA expression, the biological activity of NO was unchanged, suggesting that NOS activity was inhibited by elevated ADMA. In young, healthy humans who were exposed to high altitude (3,500 m) for the first time in an intermittent, weekly exposure regimen for 3 months developed a progressive elevation of circulating ADMA levels that significantly correlated with the elevation of haematocrit (153). In a cross-sectional study of Chilean mining workers who had been exposed to intermittent work at elevations of 4,400–4,800 m for more than 5 years, elevated ADMA levels were also significantly associated with elevated mean pulmonary artery pressure (24). Recent genetic analyses performed in our laboratory revealed significant associations of single nucleotide polymorphisms (SNPs) in the NOS III, DDAH1, AGXT2, and ARG2 genes with high altitude pulmonary hypertension (188). Specifically, individuals homozygous for the minor allele of DDAH1 SNP rs233112 had higher baseline ADMA plasma concentration but no change in the ADMA response to hypoxia (188). By contrast, homozygous carriers of the minor allele of the rs805304 SNP in the DDAH2 gene had a diminished ADMA increase during hypoxia but no difference in baseline ADMA concentration. In a parallel animal study, DDHA1 ko mice showed no difference in hypoxia-induced pulmonary arterial pressure or right ventricular morphology as compared to wild-type littermates (143). DDAH1 knockout mice, however, displayed pulmonary upregulation of DDAH2 protein during chronic hypoxia, predominantly in alveolar epithelial cells, suggesting that DDAH2 upregulation may compensate for deficient DDAH1 expression and/or activity and thereby limit the pathophysiological consequences of chronic hypoxia on pulmonary vascular NO function. To a similar point, we observed a gradual decline of SDMA in humans exposed to chronic intermittent hypoxia at altitude, which paralleled the gradual increase in ADMA as reported above (60). Homozygous carriers of AGXT2 rs37369 showed a greater reduction in plasma SDMA than carriers of the minor allele of this SNP, suggesting an upregulation of AGXT2 in hypoxia (188).

## Conclusions and Future Perspectives

Dysfunctional endothelium-dependent, NO-mediated vasodilation contributes to sustained HPV. There is accumulating evidence that elevated concentrations of the endogenous NOS inhibitor, ADMA, are involved in downregulating pulmonary vascular NO production in chronic hypoxia. Whilst studies in animal models and clinical cohort studies at high altitude are useful to dissect the molecular mechanisms of this regulation, it may have important clinical impact in understanding the pathophysiology of chronic pulmonary diseases like COPD and OSAS. Current evidence suggests that downregulation of DDAH mediates hypoxic accumulation of ADMA, but data are controversial as to which isoform is involved. Further, there may be compensatory regulation of one DDAH isoform when the other one is dysfunctional as suggested by a recent study in DDAH1 ko mice, as well as upregulation of AGXT2, as suggested by recent human studies. More studies are required to clarify the mechanism of this regulation. Information on a possible dysregulation of the L-arginine – dimethylarginine – NO pathway in chronic lung diseases like COPD, OSAS, overlap syndrome, and PAH are mostly derived from small, cross-sectional studies. Small patient numbers, heterogeneous patient populations and study designs, as well as methodological shortcomings contribute to current incertitude in this field. Large, prospective biomarker studies as well as mechanistic clinical studies in acute and chronic hypoxia using state-of-the-art methods are needed to shed light on the role of this pathway in chronic hypoxic lung diseases. This may open up new avenues for better treatment of chronic hypoxia and its pulmonary and systemic hemodynamic consequences.

## Author Contributions

JH and RB contributed equally to data acquisition and writing. Both authors agreed to the final version of the manuscript.

## Funding

This work was funded by the German Federal Ministry of Education and Research under Grant no. 01DN17046 (DECIPHER). Work by the authors was also funded by the Georg and Jürgen Rickertsen Foundation, Hamburg, Germany, the Joachim Herz Foundation, Hamburg, Germany, and the Werner Otto Foundation, Hamburg, Germany (Grant no. 02/96).

## Conflict of Interest

The authors declare that the research was conducted in the absence of any commercial or financial relationships that could be construed as a potential conflict of interest.

## Publisher's Note

All claims expressed in this article are solely those of the authors and do not necessarily represent those of their affiliated organizations, or those of the publisher, the editors and the reviewers. Any product that may be evaluated in this article, or claim that may be made by its manufacturer, is not guaranteed or endorsed by the publisher.
